# Antibacterial Effects of Er:YAG Laser Irradiation on *Candida–Streptococcal* Biofilms

**DOI:** 10.3390/life15030474

**Published:** 2025-03-16

**Authors:** Zuzanna Grzech-Leśniak, Jakub Pyrkosz, Jagoda Szwach, Patrycja Kosidło, Jacek Matys, Rafał Wiench, Magdalena Pajączkowska, Joanna Nowicka, Marzena Dominiak, Kinga Grzech-Leśniak

**Affiliations:** 1 Faculty of Medicine and Dentistry, Wroclaw Medical University, 50-367 Wroclaw, Poland; zuzanna.grzech-lesniak@student.umw.edu.pl; 2 Faculty of Medicine, Wroclaw Medical University, 50-367 Wroclaw, Poland; jakub.pyrkosz@student.umw.edu.pl (J.P.); jagoda.szwach@student.umw.edu.pl (J.S.); patrycja.kosidlo@student.umw.edu.pl (P.K.); 3 Laser Laboratory, Dental Surgery Department, Faculty of Dentistry, Wroclaw Medical University, 50-367 Wroclaw, Poland; jacek.matys@umw.edu.pl (J.M.); marzena.dominiak@umw.edu.pl (M.D.); 4 Department of Periodontal Diseases and Oral Mucosa Diseases, Faculty of Medical Sciences in Zabrze, Medical University of Silesia, 40-055 Katowice, Poland; rwiench@sum.edu.pl; 5 Department of Microbiology, Faculty of Medicine, Wroclaw Medical University, 50-367 Wroclaw, Poland; magdalena.pajaczkowska@umw.edu.pl (M.P.); joanna.nowicka@umw.edu.pl (J.N.); 6 Department of Periodontics, School of Dentistry, Virginia Commonwealth University VCU, Richmond, VA 23298, USA

**Keywords:** *Candida albicans*, *Candida glabrata*, Erbium laser, *Streptococcus mutans*

## Abstract

In contemporary dentistry, laser-based interventions have become one of the mainstays of care for patients with oral biofilm diseases, such as candidiasis, periodontal disease and peri-implantitis. The purpose of this study was to evaluate the effectiveness of Er:YAG laser (LightWalker, Ljubljana, Fotona, Slovenia) irradiation at varying irradiance levels (T1: 11.3 W/cm^2^ and T2: 120.54 W/cm^2^) on microbial viability in single- and dual-species biofilm models, focusing on *Candida albicans*, *Candida glabrata* and *Streptococcus mutans*, to address challenges in managing complex oral biofilms in clinically relevant settings. The results showed substantial microbial reduction, with *C. albicans* being the most susceptible microorganism (93–99.9%), while *C. glabrata* exhibited marked resistance at higher irradiance levels. Interestingly, *S. mutans* demonstrated varying reductions based on the biofilm composition, highlighting the influence of microbial interactions. This study concluded that the Er:YAG laser effectively reduced biofilm viability, with its efficacy depending on the microbial composition and irradiance settings. These findings highlight the need for tailored erbium laser parameters to optimize clinical outcomes, underscoring the need for individualized polymicrobial biofilm management, particularly in periodontal and peri-implant therapies.

## 1. Introduction

Microorganisms often exist within complex biofilms that facilitate multiple interactions in the mouth, which may be cooperative, mutualistic, synergistic or competitive. Fungal species such as *Candida albicans* are commonly found alongside bacteria like *Streptococcus mutans* in oral biofilms, where their interactions are synergistic. Although *S. mutans* has long been considered the primary causative agent of dental caries, recent research indicates an increased prevalence of *S. mutans* that coexists with *C. albicans* in biofilms, enhancing their virulence especially in cariogenic biofilms, advanced oral mucosal infections or peri-implantitis [1,2,3]. Bacterial–fungal interactions have become a growing area of interest due to their strong link to oral and pharyngeal diseases. The human oral cavity hosts a variety of microorganisms, including Streptococci and the yeast *Candida* spp. Studies indicate that multispecies biofilms containing these organisms can be associated with several oral health issues, including denture stomatitis, tooth decay, gum disease, endodontic complications, peri-implant infections and oral cancer [3,4,5,6,7,8,9].

Effective oral biofilm management involves regular mechanical removal through brushing and professional dental cleaning. As antibiotic and antifungal resistance increases, there is a growing need to explore advanced combined therapies and techniques, with the use of antimicrobial agents, photodynamic therapy and photobiomodulation with different wavelengths of lasers for targeted biofilm disruption [10,11,12,13,14,15,16,17,18]. Erbium lasers are used for different purpose in many fields in dentistry, including endodontics, prosthodontics, orthodontics, periodontics and oral pathology management [19,20,21,22,23,24,25,26,27]. Consistent biofilm control not only preserves oral health but also reduces the risk of systemic complications linked to oral infections [28,29,30,31].

The erbium family of lasers effectively targets fungi and bacteria through its photoacoustic and photothermal effects. When the laser interacts with water within the biofilm, it induces rapid vaporization, creating micro-explosions that mechanically disrupt the biofilm structure. These photoacoustic waves generate pressure changes, fragmenting the extracellular matrix and releasing microorganisms from the biofilm [32,33,34]. This process reduces microbial load and exposes deeper biofilm layers for further treatment or immune response. The thermal energy produced by the laser additionally damages microbial cell walls, enhancing antimicrobial efficacy [35,36,37,38,39,40]. These mechanisms make the Er:YAG laser a highly effective tool for biofilm disruption, as demonstrated in studies addressing both bacterial and fungal biofilms, such as *Streptococcus mutans* and *Candida albicans*.

The purpose of this study was to evaluate the effects of an erbium: yttrium–aluminium–garnet (Er:YAG) laser at 2940 nm on planktonic single- and dual-species cultures and biofilms composed of *Candida* spp. and *Streptococcus mutans*. Additionally, this study investigated novel low-fluence laser parameters tailored for the clinical treatment of periodontitis and peri-implant inflammation, aiming to effectively target and eradicate pathogens in mixed biofilms containing Candida spp. The null hypothesis states that Er:YAG laser irradiation has no significant antifungal effect on *Candida* spp. biofilm, meaning it neither inhibits biofilm growth nor reduces the viability of mature biofilm, regardless of the applied low-fluence and low-irradiance laser settings.

## 2. Materials and Methods

### 2.1. Sample Preparation

This study utilized clinical microorganisms of *Candida albicans*, *Candida glabrata* and *Streptococcus mutans* isolated from patients diagnosed with oral candidiasis and carious lesions (Ethical Committee Approval No: KB- 429/2024). The research protocol was developed based on a previous experiment using a neodymium laser application [41].

Samples were preserved in trypticase soy broth (Biomaxima, Lublin, Poland) at −80 °C, with each maintained in duplicate. Before experimentation, the strains were grown under optimal conditions for their optimal growth:

- *Candida* spp.: incubated aerobically on Sabouraud Dextrose Broth (Biomaxima, Lublin, Poland) at 37 °C for 48 h.
- *S. mutans*: grown on Brain Heart Infusion (BHI) agar (Biomaxima, Lublin, Poland) at 37 °C for 48 h under an atmosphere enriched with 5% CO_2_.

### 2.2. Sample Preparation Methods

To ensure better differentiation of results, two types of sample preparations were used:

(a) Planktonic cell solutions: Microbial suspensions were adjusted to a 0.5 McFarland density following these preparations:
  - Single-species cultures: 100 µL of microbial suspension (*C. albicans*, *C. glabrata* and *S. mutans*) was mixed with 900 µL of BHI broth containing 5% sucrose.
  - Mixed cultures: combination of 100 µL of each microorganism was added to 800 µL of BHI broth to create the following:
    - *C. albicans* + *S. mutans (CASM)*;
    - *C. glabrata* + *S. mutans (CGSM)*;
    - *C. albicans* + *C. glabrata (CACG)*.
(b) Biofilm formation

This study utilized two biofilm models: single-species and dual-species biofilms. The biofilms were cultivated in dark 96-well flat-bottom polystyrene plates (FL Medical, Equimed, Krakow, Poland) under controlled conditions.

- Single-species biofilm: each well received 100 µL of microorganism suspension (0.5 McFarland density) (*C. albicans*, *C. glabrata* or *S. mutans*) along with 150 µL of BHI broth containing 5% sucrose, bringing the total well volume to 250 µL per well.
- Dual-species biofilm: 100 µL of each different microorganism suspension (0.5 McFarland density) was mixed, along with 50 µL of BHI broth containing 5% sucrose, making a total volume of 250 µL.

The plates were incubated at 37 °C for 24 h, with aerobic conditions for Candida spp. and 5% CO_2_ environment for *Streptococcus mutans*. Biofilm formation was observed on both the bottom and walls of the wells.

### 2.3. Laser Application

All samples were covered with dark Eppendorf tubes (for planktonic cultures) or dark 96-well polystyrene plates (for biofilm) and irradiated using an Er:YAG dental laser handpiece (H14, LightWalker, Ljubljana, Fotona, Slovenia) equipped with a 1.3 mm diameter cylindrical sapphire tip.

**Figure 1:**
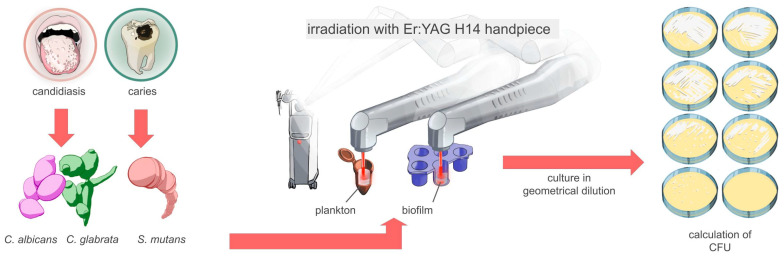
Diagram illustrating the methodology for erbium laser irradiation with H14 dental handpiece and cylindric tip of planktonic cell solutions and biofilms.

To minimize laser light scattering, dark tubes and polystyrene plates were used, ensuring that non-irradiated controls remained unaffected. This setup allowed for controlled experimental conditions to ensure reliable results. The laser handpiece was positioned 10 mm above the sample surface, either over the microbial suspension (planktonic cultures) or near the well’s edge (biofilm) (Figure 1).

#### Experimental Groups

Two distinct laser energy settings were applied: T1 (lower power): 0.15 W, 2 Hz, 30 s and 11 W/cm^2^ and T2 (higher power): 1.6 W, 40 Hz, 30 s and 120 W/cm^2^. A control group was not exposed to laser irradiation (Table 1). All experiments were performed in 2 replications. The selection of parameters for the T2 group was based on their established use in clinical applications for periodontal pocket debridement, where higher irradiance has been shown to effectively reduce bacterial loads [37,38]. These parameters were chosen as a benchmark to evaluate their efficacy compared to significantly lower irradiance levels (T1), which were included to explore whether minimal antibacterial effects could still be achieved. This comparison aimed to determine the potential of T1 settings for applications such as peri-implantitis treatment, focusing on the decontamination of implants and surrounding bone while minimizing energy output.

### 2.4. Measurement of Microbial Load

#### 2.4.1. Immediate and 24 Post-Irradiation Reduction in CFU/mL (Colony-Forming Units) (Planktonic Cultures)

Laser irradiation experiments were conducted on dark Eppendorf tubes containing single- or dual-species microbial suspensions. Immediately following irradiation, 100 µL of each sample was extracted, serially diluted in geometric progression, and 0.1 µL of the final dilution was plated onto solid BHI agar. The plates were incubated at 37 °C for 24 h under conditions matching the microbial species: aerobic incubation for Candida sp. and elevated CO_2_ atmosphere for *Streptococcus mutans*. Colony counts from the incubated plates were used to calculate CFU/mL values.

Following laser exposure, suspensions were incubated for 24 h at 37 °C. After incubation, 100 µL of each suspension was extracted, diluted serially, and 0.1 µL of the final dilution was plated onto BHI agar. These plates were incubated for another 24 h, and CFU/mL values were calculated.

The control samples were processed identically, but without laser exposure.

#### 2.4.2. Assessment of Biomass Reduction in Biofilms (Quantitative Method—CFU/mL)

Freshly prepared single- and dual-species biofilms were used for testing. To remove non-adherent cells, wells were washed three times with 0.9% NaCl solution before laser irradiation. The biofilms were then exposed to the Er:YAG laser and immediately scraped using sterile swabs. The collected biofilm material was then agitated in 0.5% saponin solution (Sigma-Aldrich, Saint Louis, MO, USA) and vortexed for 1 min to ensure biofilm cell dispersion. A 100 µL aliquot of the resulting suspension was serially diluted in geometric progression, and 0.1 µL of the final dilution was plated onto a solid growth medium for quantitative analysis (for bacterial–fungal biofilm, it was BHI agar; for CA-CG biofilm, it was after CHROMagar Candida medium (Becton Dickinson)—growth of *C. albicans* colonies appearing green; growth of *C. glabrata* colonies appearing purple). The plates were incubated at 37 °C for 24 h, with the yeast biofilms (Candida spp.) maintained under aerobic conditions, while the *Streptococcus mutans* biofilms were incubated under an elevated CO_2_ environment. Colony counts from the incubation were used to calculate CFU/mL values.

For the control group, biofilms were prepared and processed using the same protocol but were not subjected to laser irradiation.

#### 2.4.3. Biofilm Biomass Measurement (Crystal Violet Assay)

To assess biofilm biomass, a crystal violet assay was conducted. Fixed biofilms were air-dried for 45 min at room temperature. Next, 250 µL of 0.1% crystal violet solution (Chempur, Piekary Ślaskie, Poland) was added to each well, and plates were incubated for 20 min to allow staining. Excess violet was carefully removed, and wells were washed three times with distilled water, followed by an additional 20 min of air-drying. Biofilm biomass was quantified by dissolving the bound dye in 200 µL of 95% ethanol (Honeywell, Charlotte, NC, USA) and measuring at 540 nm using an ELISA reader (Biochrom Asys UVM 340; Biochrom Ltd., Fullerton, CA, USA).

### 2.5. Statistical Analysis

All statistical analyses were performed using GraphPad Prism software (version 10.0.3; GraphPad Software, San Diego, CA, USA). The Shapiro–Wilk’s test was applied to assess whether the data followed a normal distribution. Depending on the results, either parametric or non-parametric statistical tests were used for analysis. For comparisons involving multiple groups, data that met the normality criteria were analyzed using one-way ANOVA, followed by Turkey’s post hoc test. If the normality assumption was violated, the Kruskal–Wallis test was employed, with Dunn’s post hoc test for pairwise comparisons. For two-group comparisons, Student’s *t*-test was used when data were normally distributed, while Mann–Whitney U test was applied to non-normally distributed data. Results were reported as mean ± standard deviation (SD), and statistical significance was set at *p* < 0.05. Additionally, correlation analysis was performed to investigate potential association between biofilm reduction and laser energy settings, using Pearson correlation for normally distributed data and Spearman correlation for non-parametric data. All tests were carried out at 95% confidence interval (CI) to ensure reliability of the findings.

## 3. Results

### 3.1. Microbial CFU Reduction in Single-Species Planktonic Cultures

This study evaluated the reduction in colony-forming units (CFU/mL) following Er:YAG laser irradiation in single-species planktonic cultures. The most significant reduction was observed for *Candida glabrata,* with mean CFU/mL of 92.6% ± 4.2% at the T1 settings and 88.5% ± 4.7% at the T2 settings after 24 h.

However, for *C. albicans* and *C. glabrata*, neither laser setting (T1 vs. T2) nor the time of assessment (directly after irradiation (DAI) vs. 24 h after irradiation (24AI)) demonstrated a significantly greater reduction in microbial count (*p* > 0.05).

Directly after laser application, *Streptococcus mutans* showed a significantly greater reduction with T1 settings compared to T2, at 80.0% ± 6.1% and 41.4% ± 4.2%, respectively (*p* < 0.05). Furthermore, a significantly higher reduction efficiency was observed directly after laser application compared to the 24 h follow-up for both laser settings (T1 and T2) (*p* < 0.05) (Table 2).

### 3.2. Microbial CFU Reduction in Dual-Species Planktonic Cultures

The highest reduction in microbial count was observed for *Candida albicans* (C.a.) in the CASM mixture, at 81.5% ± 2.3% and 71.2% ± 1.8% when measured directly after irradiation (DAI) and 24 h after laser application (24AI), respectively. Moreover, the reduction measured DAI was significantly higher than at 24AI (*p* < 0.05). Additionally, Er:YAG laser irradiation using the T1 settings resulted in significantly greater microbial count reduction compared to the T2 settings at both time points (*p* < 0.05).

In contrast, *S. mutans* in the CASM mixture exhibited significantly greater CFU reduction 24 h after Er:YAG laser irradiation at the T1 settings, and significantly lower reduction when measured at the T2 settings (*p* < 0.05). Furthermore, in the CGSM mixture, *Streptococcus mutans* showed greater CFU reduction 24 h after the Er:YAG laser intervention (T1: 58.0% ± 19.0%; T2: 18.8% ± 3.0%) compared to directly after intervention (T1: 47.5% ± 31.8%; T2: 0.0% ± 0.0%) (*p* < 0.05).

*Candida glabrata* in the CACG mixture demonstrated significantly greater reduction at the T1 settings compared to T2, with reductions of 36.2% ± 3.0% and 3.2% ± 4.5%, respectively (*p* < 0.05) (Table 3).

### 3.3. Microbial CFU Reduction in Single-Species Biofilm Cultures

In single-species biofilms, CFU reduction ranged from 98.4% ± 1.3% for *C. glabrata* to 56.1% ± 8.6% for *C. albicans* under T2 laser parameters.

However, significant differences were observed only for *C. albicans* between the T1 (95.3% ± 2.8%) and T2 laser settings (56.1% ± 8.6%) (*p* < 0.05) (Table 4).

### 3.4. Microbial CFU Reduction in Dual-Species Biofilm Cultures

Higher mean reductions in %CFU were observed in dual-species biofilm cultures at higher irradiance (T2) compared to the lower settings (T1). For T2, reductions were as follows: *Candida albicans* (CACG: 99.9% ± 0.1%, CASM: 99.9% ± 0.1%), *Candida glabrata* (CACG: 98.8% ± 0.6%, CGSM: 100.0% ± 0.0%), and *Streptococcus mutans* (CASM: 99.9% ± 0.0%, CGSM: 100.0% ± 0.0%). For T1, reductions were as follows: *Candida albicans* (CACG: 99.0% ± 0.2%, CASM: 96.5% ± 0.2%), *Candida glabrata* (CACG: 79.9% ± 9.9%, CGSM: 39.1% ± 8.8%), and *Streptococcus mutans* (CASM: 96.9% ± 1.8%, CGSM: 47.3% ± 10.4%).

Significantly greater %CFU reductions were observed with T2 compared to the T1 settings for *C. albicans* in the CACG and CASM mixtures, *C. glabrata* in the CGSM mixture, and *S. mutans* in the CGSM mixture (*p* < 0.05) (Table 5).

### 3.5. Biofilm Biomass Reduction (Crystal Violet Assay)

In the *C. albicans–S. mutans* (CASM) biofilm, a significantly higher reduction in biomass was observed at the T1 settings (33.7% ± 1.1) compared to the T2 settings (4.8% ± 5.2) (*p* = 0.016), indicating that higher laser power (T2) was less effective in reducing biofilm biomass compared to lower power (T1). Similarly, the *C. glabrata–S. mutans* (CGSM) biofilm exhibited a significantly greater reduction at T1 (21.8% ± 3.1) compared to the T2 settings (2.15% ± 3.0) (*p* = 0.023), further suggesting decreased efficiency at higher laser power. Additionally, in the *C. albicans–C. glabrata* (CACG) biofilm, no statistically significant reduction was observed between the two assessed settings: T1 (16.4% ± 12.1) and T2 (0% ± 0) (*p* = 0.196). The ANOVA results confirmed that the overall differences between the groups were not statistically significant (*p* = 0.192 for T1 and *p* = 0.380 for T2). (Table 6)

## 4. Discussion

Our research underscores the critical importance of addressing yeast biofilms due to the significant interactions between *Candida* spp. and bacterial species, which are important in the formation and progression of biofilms and associated oral diseases. These synergistic interactions exacerbate conditions such as caries, periodontal diseases and peri-implantitis. Among the microorganisms evaluated, *Candida albicans* demonstrated the greatest sensitivity to biofilm reduction. The key findings demonstrated that Er:YAG laser irradiation could inhibit biofilm growth even at relatively low energy and fluence (40 mJ; 3 J/cm^2^), while also significantly reducing mature Candida spp. biofilms in combination with *S. mutans*. A reduction of 95.3% ± 2.8% in single-species biofilms and reductions between 96.5% ± 0.2% and 99.9% ± 0.1% in dual-species biofilms were achieved for this species using Er:YAG laser irradiation. A significantly greater decrease in microbial count was observed after laser irradiation, particularly 24 h post-irradiation, for both irradiance parameters (T1: 11 W/cm^2^ and T2: 120 W/cm^2^). *Candida glabrata*, while generally more resistant to physical and chemical treatments, showed a reduction of 98.4% ± 1.3% in single-species biofilms under the T2 settings. Conversely, in dual-species biofilms, *C. glabrata* demonstrated distinctive response patterns, particularly at higher irradiance settings (T2), where the reduction in microbial load was significantly lower, reaching only 3.2% ± 4.5%. These findings highlight the Er:YAG laser’s effectiveness in reducing microbial loads, particularly in biofilms containing *Candida albicans* and mixed-species compositions. However, the results also emphasize the importance of adjusting laser settings to the specific microbial composition within the biofilm to achieve optimal clinical outcomes.

The findings of this study indicate that lower-power Er:YAG laser settings (T1) were more effective in reducing biofilm biomass compared to the higher-power settings (T2). This observation is counterintuitive, as higher power typically correlates with greater antimicrobial effects. However, multiple factors, including heat distribution, microbial adaptive responses, and biofilm structural alterations, may explain this phenomenon.

One possible explanation for the greater biofilm reduction observed at the T1 settings is related to thermal energy dissipation. Lower power settings deliver energy more gradually, potentially causing a sustained thermal effect that destabilizes microbial cells and biofilm architecture without leading to excessive heat accumulation. In contrast, higher power settings (T2) may lead to rapid energy deposition, generating a more intense but localized heating effect. Excessive heat can induce protective mechanisms within microbial communities, including heat shock responses, which may promote cellular survival rather than eradication. Some studies have suggested that abrupt thermal stress triggers microbial adaptation strategies, leading to increased biofilm resilience and reducing overall effectiveness at higher energy settings. The architecture of mixed-species biofilms plays a crucial role in their susceptibility to laser irradiation. Er:YAG lasers primarily interact with water molecules, and their effectiveness depends on the hydration level of the biofilm matrix. Under the T1 settings, laser energy may be optimally absorbed by the biofilm water content, leading to progressive structural disruption without immediate collapse. However, under the T2 settings, rapid vaporization could result in biofilm contraction, where the surface layers shrink, limiting laser penetration into deeper biofilm regions. This effect could shield embedded microbial cells from complete eradication, explaining the reduced biofilm biomass elimination at higher power settings.

These findings emphasize the importance of adjusting laser parameters based on biofilm composition and structure. While higher laser power (T2) might be beneficial for mechanical debridement, the controlled thermal effects at lower power (T1) appear to enhance microbial reduction, particularly in Candida–Streptococcus dual-species biofilms.

Previous research by our group, reported in Grzech-Leśniak et al. [41], using Nd:YAG laser at low energy settings has demonstrated a significant reduction in pathogens, often exceeding 95% immediately after irradiation (DAI), depending on the fluence and parameters used. The Nd:YAG laser, with its 1064 nm wavelength, penetrates deeper into biofilms compared to the Er:YAG laser (2940 nm), which primarily affects surface biofilm layers due to its high absorption in water. This superior penetration depth of the Nd:YAG laser is particularly advantageous in disrupting deeper biofilm layers, where microorganisms are often more resistant to conventional treatments. A study from 2019 [42] demonstrated that high microbial reductions were achieved in single-species biofilms of *Candida albicans* and *Streptococcus mutans* using the Nd:YAG laser with low-level laser settings, underscoring its effectiveness in eliminating pathogens beyond the surface layer. By contrast, our study observed that while the Er:YAG laser was effective in reducing microbial loads, particularly for *C. albicans*, its efficacy was reduced in dual-species biofilms, especially those with *C. glabrata*, under higher irradiance settings (T2). These differences align with the findings of Grzech-Leśniak et al. (2024) [41], which emphasize the importance of laser parameter optimization to address biofilm complexity. This research builds on these insights, showing that while erbium lasers have clinical relevance, they require tailored settings for complex biofilms, where neodymium lasers may excel in certain contexts.

Er:YAG lasers, known for their photothermal and photoacoustic effects, have shown significant efficacy in biofilm disruption by targeting water molecules in the extracellular matrix and inducing cavitation. In clinical applications, these mechanisms have proven effective in disrupting biofilms through photothermal effects and cell wall disruption on tooth surfaces, root canals, peri-implant areas and other oral tissues [43,44,45,46,47,48,49]. Despite the superior depth of penetration offered by Nd:YAG lasers, Er:YAG lasers have demonstrated high efficacy in biofilm reduction, particularly in dual-species biofilms. Their ability to disrupt biofilm structures mechanically, in addition to their antimicrobial effects, makes them a valuable tool in non-surgical periodontal therapy. However, Nd:YAG lasers’ ability to reach deeper layers suggests that they may be more effective in cases where subgingival biofilms or peri-implant infections require a more extensive decontamination approach. Our findings reveal that the Er:YAG laser achieved substantial reductions in microbial counts for *C. albicans*, with reductions exceeding 99.9% in some dual-species biofilms, especially for *C. albicans–S. mutans* (CASM) and *C. albicans–C. glabrata* (CACG) biofilms. However, *Candida glabrata* exhibited notable resistance at higher irradiance levels (T2) for dual-species planktonic cultures and at lower irradiance (T1) for dual-species biofilm cultures, highlighting the species-specific nature of biofilm resilience. Comparative studies by Nagahashi et al. (2022) and Terlep et al. (2023) have demonstrated that Er:YAG lasers achieve significant biofilm reductions, particularly in constrained geometries mimicking periodontal pockets, through photoacoustic streaming and cavitation [46,47]. While these studies confirm the efficacy of Er:YAG lasers in biofilm disruption, our results underscore the need for tailored irradiance settings to address species-specific resistance patterns, particularly in complex biofilms involving multiple microorganisms [48].

CO_2_ lasers have also demonstrated notable dual effects in biofilm management, disrupting biofilm layers through photothermal mechanisms while inducing microbial adaptation responses, such as stress-related gene expression and DNA methylation changes [50]. Studies by Cohen et al. (2014) and Gryzińska et al. (2023) highlighted the antibacterial and antifungal potential of CO_2_ lasers, particularly against *Candida albicans* and *Streptococcus mutans* biofilms [50,51]. Cohen et al.’s research on CO_2_ laser effects demonstrated their antibacterial efficacy through photothermal mechanisms, effectively penetrating biofilm layers and altering enamel properties to improve resistance to acid attacks, suggesting their potential for caries prevention [50]. Gryzińska et al. further explored epigenetic adaptations in *C. albicans*, showing that physical factors such as laser exposure can increase DNA methylation levels, potentially enhancing biofilm resilience through gene regulation [51]. Compared to Er:YAG lasers, CO_2_ lasers offer deeper penetration, altering enamel properties to enhance resistance to acid attacks, making them promising tools for caries prevention [52,53,54]. Additionally, autofluorescence studies, such as those by Wiench et al. (2024), have demonstrated diagnostic advantages, with *Candida albicans* exhibiting stronger autofluorescence properties under 405 nm light compared to *Candida glabrata* [55]. These findings suggest that autofluorescence-guided laser therapies could enhance the precision of biofilm management strategies. Studies have demonstrated that in mixed-species biofilms, erbium lasers may provide better microbial reductions without compromising enamel or dentin integrity, as shown in research focusing on dental applications [46,56,57,58,59]. These results complement studies using Nd:YAG and Er:YAG lasers, highlighting the diagnostic and therapeutic potential for managing Candida infections.

The interaction between *S. mutans* and *Candida* spp. increases resistance to conventional treatments, emphasizing the need for laser-based therapies that penetrate biofilm layers effectively. In the current study, *Streptococcus mutans* in single-species planktonic cultures exhibited an 80.0% ± 6.1% (*p* < 0.05) reduction immediately after laser irradiation, even with low power settings. However, in dual-species cultures involving *C. albicans* or *C. glabrata*, higher irradiance and a longer duration (24 h) were required to achieve substantial CFU reductions (99.0% to 100.0%, *p* < 0.05). According to the National Committee for Clinical Laboratory Standards (NCCLS) guidelines, an antimicrobial treatment achieves ≥ 3 log₁₀ CFU/mL reduction, equivalent to a 99.9% reduction. In this study, certain conditions resulted in microbial reductions exceeding 99.9%, suggesting potential bactericidal/fungicidal activity. However, there were cases where reductions were lower, highlighting the need for further optimization of laser parameters to enhance antimicrobial efficacy [60]. These findings align with previous studies using Nd:YAG lasers, where better reductions in *C. glabrata* were achieved immediately after irradiation (DAI), contrasting with Er:YAG lasers, which required more time and higher parameters [41]. In this study, *C. glabrata* showed significant reductions only under the T1 settings (36.2% ± 3.0%) and markedly lower reductions under the T2 settings (3.2% ± 4.5%). These results underscore the importance of tailoring laser parameters and integrating advanced imaging techniques to optimize biofilm management and enhance clinical relevance. Together, these methodologies highlight the importance of tailoring experimental designs to the research context, balancing simplicity with providing translational value for clinical use [61,62,63]. Our findings indicate that lower laser power (T1) was more effective in reducing biofilm biomass compared to higher power (T2). This observation aligns with studies utilizing the Er:YAG laser, which has demonstrated significant efficacy in biofilm removal. For instance, Polak et al. found that the Er:YAG laser effectively removed multispecies biofilms from titanium surfaces, outperforming traditional methods such as hand curettes and ultrasonic devices, even at low energy settings [64]. Additionally, studies have reported enhanced bacterial and lipopolysaccharide clearance, as well as improved human gingival fibroblast adhesion, on titanium and zirconia disc surfaces [65,66,67]. Similarly, AlMoharib et al. reported that Er:YAG laser treatment significantly reduced the bacterial biofilm on titanium discs, achieving comparable results to mechanical debridement tools like titanium brushes and carbon fiber curettes [68]. These studies suggest that Er:YAG lasers are effective in biofilm removal at lower energy settings, supporting our findings that higher laser power does not necessarily enhance biofilm reduction and may even be less effective.

Despite the promising findings, this study has certain limitations. The in vitro nature of the experiments, using controlled biofilm models, may not fully replicate clinical conditions, where factors such as saliva flow, immune responses and biofilm complexity are more pronounced. Additionally, the observed resistance patterns of *Candida glabrata* at higher irradiance levels underscore the need for further research to understand the underlying mechanisms and to optimize laser parameters for species-specific efficacy. Another limitation of this study is the low number of replications, which may impact the reproducibility of the findings and necessitate further validation through larger-scale studies. Future studies should explore the integration of advanced imaging techniques, such as autofluorescence-guided therapies (an innovative diagnostic approach) and scanning electron microscopy (SEM) imaging, to assess biofilm structure and surface modifications after laser treatment and investigate the combined use of lasers with photosensitizers to enhance treatment outcomes. Incorporating metabolic assays and in vivo models could provide deeper insights into the effects of laser irradiation on biofilm dynamics. Furthermore, expanding research to include diverse biofilm compositions and clinical scenarios will help validate the translational value of these findings, ultimately contributing to improved protocols for managing biofilm-associated infections.

## 5. Conclusions

The findings demonstrate that laser settings and time points have a significant impact on microbial reduction in both *Candida* spp. biofilms and planktonic cultures, depending on the microorganism and experimental conditions. Higher laser settings (T2) generally resulted in greater CFU reductions, particularly in double-species biofilms. However, certain species like *C. glabrata* and *S. mutans* exhibited unique responses, depending on the laser settings and assessment time, highlighting the importance of tailored approaches for biofilm management.

## Figures and Tables

**Table 1:** Control and test groups with the laser parameters used in this study.

Control Group	Test Group (T1)	Test Group (T2)
No laser irradiation	Power: 0.15 W	Power: 1.6 W
	Frequency: 2 Hz	Frequency: 40 Hz
	Tip diameter: 1.3 mm	Tip diameter: 1.3 mm
	Irradiation time: 30 s	Irradiation time: 30 s
	Pulse duration: 300 µs	Pulse duration: 300 µs
	Energy: 75 mJ	Energy: 40 mJ
	Fluence: 5.65 J/cm^2^	Fluence: 3.01 J/cm^2^
	Irradiance: 11.3 W/cm^2^	Irradiance: 120.54 W/cm^2^

**Table 2:** Percentage reduction in CFU/mL for all single-species planktonic cultures.

Microorganism	Time	Handpiece	Mean %CFU Reduction (SD)		*p*-Value T1 vs. T2
			T1	T2	
*Candida albicans* (C.a.)	DAI	H14	58.4% (±3.9%)	60.9% (±12.2)	0.804
	24AI		69.2% (±2.0)	79.4% (±11.5)	0.340
	*p*-value DAI vs. 24AI		0.072	0.258	x
*Candida glabrata* (C.g.)	DAI	H14	77.1% (±6.2)	62.2% (±4.8)	0.116
	24AI		92.6% (±4.2)	88.5% (±4.7)	0.454
	*p*-value DAI vs. 24AI		0.101	0.032	x
*Streptococcus mutans* (S.m.)	DAI	H14	80.0% (±6.1)	41.4% (±4.2)	0.018
	24AI		0.0% (±0.0)	0.0% (±0.0)	0.293
	*p*-value DAI vs. 24AI		0.003	0.005	x

DAI—directly after irradiation, 24AI—24 h after irradiation, T1—lower power, T2—higher power, C.a.—*Candida albicans*, C.g.—*Candida glabrata*, S.m.—*Streptococcus mutans*, %CFU—percentage reduction in the number of colony-forming units (CFUs), SD—standard deviation.

**Table 3:** Percentage reduction in CFU/mL for dual-species planktonic cultures.

Microorganisms	Mixture	Time	Handpiece	Mean %CFU Reduction (SD)		*p*-Value T1 vs. T2
				T1	T2	
*Candida albicans* (C.a.)	CACG	DAI	H14	8.7% (±12.2%)	8.4% (±11.8%)	0.982
		24AI		26.3% (±14.4%)	55.1% (±21.3%)	0.254
		*p*-value DAI vs. 24AI		0.318	0.113	x
	CASM	DAI	H14	81.5% (±2.3%)	29.9% (±14.4%)	0.037
		24AI		71.2% (±1.8%)	10.0% (±14.1%)	0.026
		*p*-value DAI vs. 24AI		0.040	0.296	x
*Candida glabrata* (C.g.)	CGCA	DAI	H14	33.0% (±21.1%)	19.6% (±17.5%)	0.559
		24AI		36.2% (±3.0%)	3.2% (±4.5%)	0.013
		*p*-value DAI vs. 24AI		0.851	0.327	x
	CGSM	DAI	H14	18.1% (±1.9%)	10.8% (±13.0%)	0.517
		24AI		66.3% (±16.3%)	34.7% (±11.5%)	0.155
		*p*-value DAI vs. 24AI		0.054	0.192	x
*Streptococcus mutans* (S.m.)	CASM	DAI	H14	0.0% (±0.0%)	44.2% (±2.6%)	0.002
		24AI		20.0% (±1.6%)	1.7% (±2.3%)	0.012
		*p*-value DAI vs. 24AI		0.003	0.003	x
	CGSM	DAI	H14	47.5% (±31.8%)	0.0% (±0.0%)	0.169
		24AI		58.0% (±19.0%)	18.8% (±3.0%)	0.103
		*p*-value DAI vs. 24AI		0.033	0.012	x

DAI—directly after irradiation; 24AI—24 h after irradiation; T1—lower laser power; T2—higher laser power; C.a.—*Candida albicans*; C.g.—*Candida glabrata*; S.m.—*Streptococcus mutans*; CACG—mixture of *C. albicans* + *C. glabrata*; CASM—mixture of *C. albicans* + *S. mutans*; CGSM—mixture of *C. glabrata* + *S. mutans*; %CFU—percentage reduction in the number of colony-forming units (CFU); SD—standard deviation.

**Table 4:** Summary of CFU/mL reduction in single-species biofilms.

Microorganisms	Time	Handpiece	Mean CFU Reduction % (SD)		*p*-Value T1 vs. T2
			T1	T2	
*Candida albicans* (C.a.)	DAI	H14	95.3% (±2.8%)	56.1% (±8.6%)	0.026
*Candida glabrata* (C.g.)	DAI	H14	65.5% (±25.6%)	98.4% (±1.3%)	0.212
*Streptococcus mutans* (S.m.)	DAI	H14	80.4% (±21.9%)	96.1% (±5.3%)	0.430

DAI—directly after irradiation at 24 h of growth, T1—lower power, T2—higher power, C.a.—*Candida albicans*, C.g.—*Candida glabrata*, S.m.—*Streptococcus mutans*, %CFU—percentage reduction in the number of colony-forming units (CFUs), SD—standard deviation.

**Table 5:** Summary of CFU reduction in dual-species biofilm cultures.

Microorganisms	Mixture	Time	Handpiece	Mean Reduction in %CFU (SD)		*p*-Value T1 vs. T2
				T1	T2	
*Candida albicans* (C.a.)	CACG	DAI	H14	99.0% (±0.2%)	99.9% (±0.1%)	0.030
	CASM	DAI	H14	96.5% (±0.2%)	99.9% (±0.1%)	0.002
*Candida glabrata* (C.g.)	CACG	DAI	H14	79.9% (±9.9%)	98.8% (±0.6%)	0.114
	CGSM	DAI	H14	39.1% (±8.8%)	100.0% (±0.0%)	0.010
*Streptococcus mutans* (S.m.)	CASM	DAI	H14	96.9% (±1.8%)	99.9% (±0.0%)	0.147
	CGSM	DAI	H14	47.3% (±10.4%)	100.0% (±0.0%)	0.019

DAI—directly after irradiation at 24 h of growth; T1—lower-power laser setting; T2—higher-power laser setting; C.a.—*Candida albicans*; C.g.—*Candida glabrata*; S.m.—*Streptococcus mutans*; CACG—mixture of *C. albicans* + *C. glabrata*; CASM—mixture of *C. albicans* + *S. mutans*; CGSM—mixture of *C. glabrata* + *S. mutans*; %CFU—percentage reduction in the number of colony-forming units (CFUs); SD—standard deviation.

**Table 6:** Percentage biomass reduction using the crystal violet method 6.9 (3.7) 11.0 (1.9) 0(0).

Mixture of Microorganisms	Reduction Biomass in %		T1 vs. T2 *p*-Value
	T1 (SD)	T2 (SD)	
CASM	33.7 (1.1)	4.8 (5.2)	*p* = 0.016
CGSM	21.8 (3.1)	2.15 (3.0)	*p* = 0.023
CACG	16.4 (12.1)	0 (0)	*p* = 0.196
ANOVA:	*p* = 0.192	*p* = 0.438	×

SD—standard deviation; CASM—*C. albicans* + *S. mutans*; CGSM—*C. glabrata* + *S. mutans*; CACG—*C. albicans* + *C. glabrata*.

## Data Availability

Data are contained within the article.
